# Revisiting proton therapy for adenoid cystic carcinoma: a case worth making

**DOI:** 10.3389/fonc.2026.1979803

**Published:** 2026-10-12

**Authors:** Astha Rohit, Adam Holtzman, James Bates, Scott Lester, Roi Dagan, Barbara Vischioni, Ester Orlandi, Maria Bonora, Amy Tien Yee Chang, Rohan Deraniyagala, Martin Bues, Steven Frank, Samir H. Patel, Atsushi Musha, Tatsuya Ohno, Nancy Lee, Lisa McGee

**Affiliations:** 1 Drexel University College of Medicine, Philadelphia, PA, United States; 2 Mayo Clinic in Florida Department of Radiation Oncology, Jacksonville, FL, United States; 3 University of North Carolina Department of Radiation Oncology, Chapel Hill, NC, United States; 4 Mayo Clinic Minnesota Department of Radiation Oncology, Rochester, MN, United States; 5 University of Florida Proton Therapy Institute, Jacksonville, FL, United States; 6 Radiation Oncology Unit, National Center for Oncological Hadrontherapy (CNAO), Pavia, Italy; 7 Hong Kong Sanitorium Hospital, Comprehensive Oncology Centre, Hong Kong, Hong Kong SAR, China; 8 Corewell Health, Royal Oak, MI, United States; 9 Mayo Clinic Arizona, Scottsdale, AZ, United States; 10 Department of Radiation Oncology, The University of Texas MD Anderson Cancer Center, Houston, TX, United States; 11 Gunma University Heavy Ion Medical Center, Gunma, Japan; 12 Memorial Sloan Kettering Cancer Center, New York, NY, United States

**Keywords:** adenoid cystic carcinoma (ACC), carbon-ion therapy, particle therapy, proton beam therapy (PBT), salivary gland cancer (SGC)

## Abstract

**Background:**

Adenoid cystic carcinoma (ACC) is a rare malignancy characterized by indolent growth, frequent perineural invasion, and a propensity for locally advanced disease involving the skull base. High-dose radiation to complex target volumes must be balanced with minimizing exposure to adjacent organs at risk, making particle therapy attractive. Proton beam therapy (PBT) and carbon ion radiotherapy (CIRT) provide favorable dose distributions compared with photon-based radiotherapy, while CIRT offers additional theoretical radiobiologic advantages through its higher linear energy transfer and relative biological effectiveness. However, whether these properties translate into superior clinical outcomes remains uncertain.

**Methods:**

This perspective reviews physical and radiobiologic characteristics of PBT and CIRT and evaluates contemporary clinical evidence supporting their use in ACC. Available studies demonstrate favorable local control, overall survival, and toxicity outcomes with both modalities but have not established a clear clinical advantage of CIRT over modern PBT. Interpretation is limited by heterogeneous patient populations, treatment techniques, fractionation schedules, institutional practices, follow-up, and the absence of prospective data. Additionally, differences in infrastructure, cost, geographic availability, and reimbursement influence treatment selection and patient access.

**Results:**

Current evidence supports PBT as an efficacious and broadly applicable treatment option for ACC, particularly for complex skull base and perineural target volumes. Future research should prioritize comparative dosimetry, refinement of biologic dose modeling, multicenter clinical outcomes, contemporary cost-effectiveness analyses, and patient selection. Expanding equitable access to particle therapy should remain a parallel objective.

**Conclusion:**

Current literature supports PBT in the treatment of ACC. Reported treatment outcomes are comparable to CIRT.

## Introduction

Adenoid cystic carcinoma (ACC) is a rare salivary gland malignancy, most commonly in the head and neck (HN), characterized by indolent growth, perineural invasion (PNI), and late hematogenous dissemination, commonly to the lungs. Its gradual symptomatic onset frequently delays diagnosis, resulting in locally advanced disease involving the skull base or other critical structures (1–13). Consequently, nearly one-third of patients present with unresectable disease or require treatment associated with substantial functional or cosmetic morbidity (14). Complete surgical resection followed by adjuvant radiation therapy (RT) remains the standard of care for resectable disease, whereas definitive RT is preferred when surgery is not feasible (15).

Although ACC was historically considered radioresistant, advances in imaging, target delineation, treatment planning, and image guidance have enabled conformal dose escalation while minimizing dose to adjacent organs at risk (OARs) (5, 6, 8, 9, 16–19). Because ACC commonly spreads along cranial nerves (CNs) to the skull base and lies in close proximity to critical neurologic and sensory structures, achieving durable local control (LC) requires high radiation doses to targets, while providing safe radiation doses to adjacent critical organs at risk (OARs) to limit toxicity (7, 12, 19, 20). These challenges have made particle therapy (PT) an attractive treatment approach.

The development of (PT) for ACC has paralleled advances in radiobiology and treatment technology. Early experiences with fast neutron therapy (FNT) demonstrated improved LC compared with historical X-ray-based RT (XRT) but were limited by substantial toxicity and restricted availability (5, 6, 17). Proton beam therapy (PBT) was introduced clinically for ACC in the early 1990s, followed shortly thereafter by carbon ion radiotherapy (CIRT) in Japan (21, 22). Both modalities provide superior dose conformity, a Bragg peak with rapid distal dose falloff, and reduced integral dose to surrounding tissues, making them well suited for tumors involving the skull base and perineural pathways (6, 20, 23–27). Carbon ions also possess higher linear energy transfer (LET) and relative biological effectiveness (RBE), generating more complex DNA damage and providing a theoretical advantage for relatively radioresistant tumors such as ACC (14, 28–30). However, whether these radiobiologic advantages translate into superior clinical outcomes compared with modern PBT remains uncertain.

As PT becomes increasingly available worldwide, defining the relative roles of PBT and CIRT has important implications for patient selection, resource utilization, accessibility, and future clinical trial design (31). In this perspective, we review the physical and radiobiologic principles of each modality, summarize the available clinical evidence, and examine practical considerations, including dosimetry, cost, and access, to evaluate whether current evidence supports a meaningful clinical advantage of CIRT over PBT.

### Physical properties (LET and RBE)

The principal distinction between PBT and CIRT lies in their radiobiologic properties. Although both modalities provide highly conformal dose distributions with reduced integral dose to normal tissues, differences in particle mass, charge, LET, and RBE produce distinct biologic effects.

Protons are light, positively charged particles with low-to-intermediate LET and a clinically assigned RBE of 1.1. Although this fixed value is used for treatment planning, experimental studies have demonstrated that proton RBE increases near the distal edge of the Bragg peak, where LET is greatest, suggesting that RBE may exceed 1.1 in selected regions (32). In contrast, carbon ions have greater mass and charge, resulting in higher LET, narrower lateral penumbra, and an RBE typically ranging from 2 to 5, depending on the radiobiologic model used (14, 28–30, 33). These properties produce more complex, less reparable DNA damage and reduce dependence on oxygen-mediated free radical formation, providing a theoretical advantage for hypoxic and relatively radioresistant tumors such as ACC (28, 29). From a radiobiological perspective, moreover, the use of CIRT for ACC management may be supported by immunological, molecular, and clinicopathological considerations.

Despite favorable radiobiologic characteristics, several factors impede the clinical demonstration of CIRT superiority. Carbon ion RBE is model dependent, and variations in treatment planning systems, prescription doses, and fractionation schedules complicate comparisons across institutions. Nuclear fragmentation beyond the Bragg peak deposits a low-dose “fragmentation tail” distal to the target, introducing uncertainty in biologic dose estimation, particularly when tumors abut critical organs at risk (28). Most importantly, prospective comparative studies demonstrating superior oncologic outcomes with CIRT over modern PBT are lacking, suggesting that treatment planning, target coverage, and patient selection remain major determinants of clinical outcomes (34).

### Dosimetric considerations

PNI is a defining feature of ACC and presents a major challenge in RT planning. Tumor spread frequently follows named CNs to the skull base, requiring long, narrow target volumes adjacent to critical OARs, including the brainstem, optic apparatus, temporal lobes, cochlea, oral cavity, and mandible (35). Consequently, successful treatment requires both comprehensive target coverage and meticulous OAR sparing. Gross PNI is typically treated to definitive doses (e.g., 70 Gray (RBE) (Gy (RBE)) in 2 Gy (RBE) fractions or 74.4 Gy (RBE) in 1.2 Gy (RBE) twice-daily fractions with photons or PBT), while proximal CN pathways are commonly included in elective target volumes even in the absence of radiographic PNI because of the high incidence of microscopic neural spread (35).

Both PBT and CIRT provide dosimetric advantages over XRT through improved conformality and reduced integral dose, making them particularly well suited for ACC (36, 37). However, direct dosimetric comparisons between PBT and CIRT are lacking. Centers such as Heidelberg and CNAO have adopted mixed-beam approaches combining photons or protons with a carbon ion boost for elective neck or extensive perineural volumes to mitigate uncertainties associated with large CIRT target volumes (38, 39).

Carbon ions may improve dose localization because of their narrower lateral penumbra and higher LET, whereas PBT may offer greater flexibility for elongated, irregular target volumes following CNs to the skull base. Although clinicians at centers offering both modalities have suggested planning advantages for PBT in extensive neural tracking, these observations remain anecdotal and have not been validated in comparative planning studies. In addition, unlike PBT, which generally follows conventional fractionation schedules, CIRT is commonly delivered using hypofractionated regimens (e.g., 64 Gy (RBE)/16 fractions) (40).

### Clinical outcomes

Contemporary studies consistently demonstrate excellent LC and overall survival (OS) with both PBT and CIRT, generally exceeding historical outcomes reported with XRT (34). These favorable results make PT an attractive option, particularly for patients with unresectable ACC involving the skull base who require definitive RT (**Figure 1**). Despite the favorable radiobiologic properties of carbon ions, current evidence has not demonstrated superior LC or OS with CIRT compared with PBT (34). Interpretation of comparative outcomes remains limited by differences in patient populations, treatment techniques, institutional experience, follow-up duration, and the absence of randomized data. Results from the ongoing phase III ETOILE trial are therefore eagerly anticipated.

**Figure 1:**
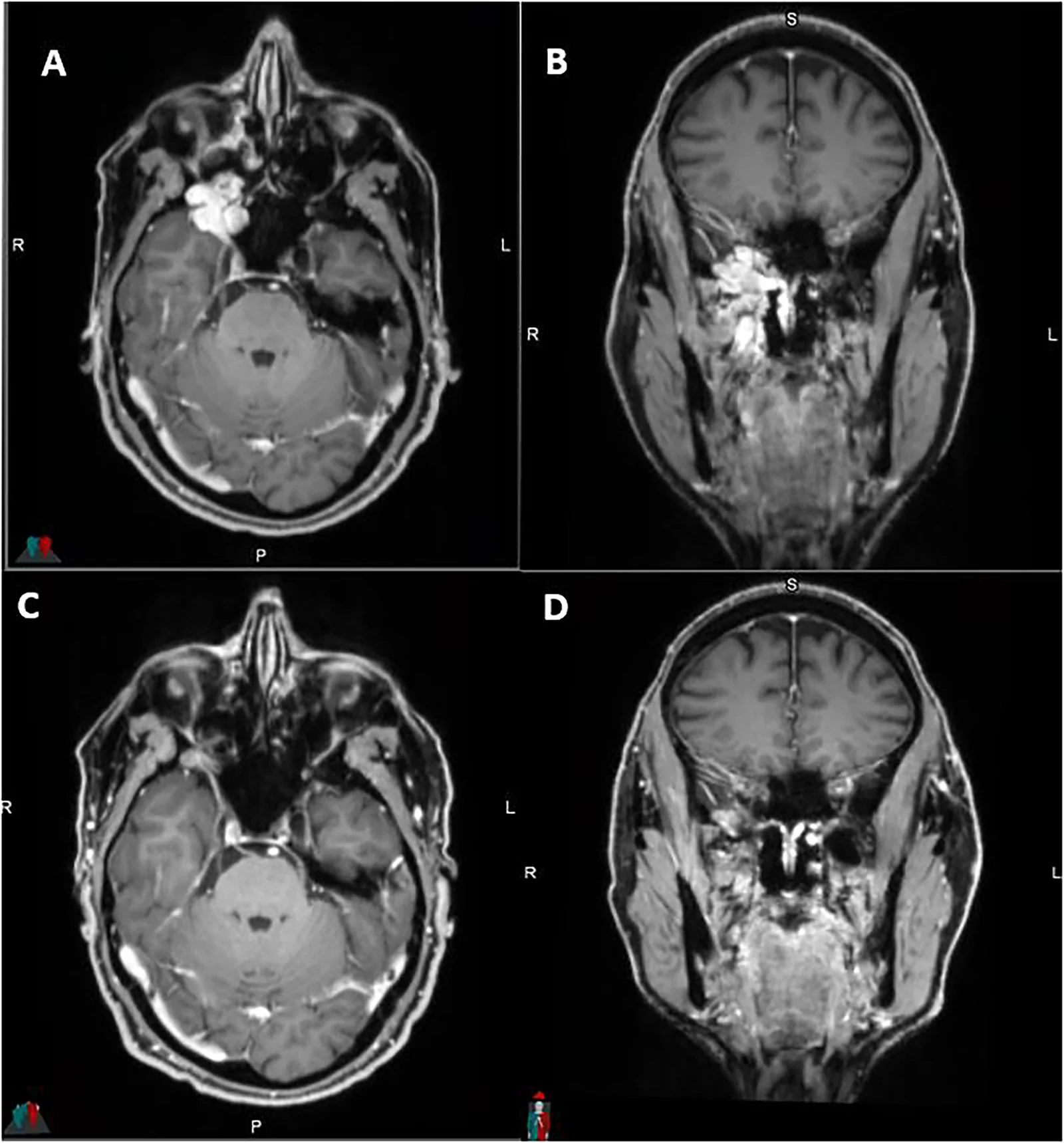
68 year-old male with a stage IVB (cT4b N0 M0), unresectable ACC of the right (R) ethmoid sinus with PNI in the R skull base. Pre-treatment axial **(A)** and coronal **(B)** T1-FS contrast-enchanced MRI. One-year post-therapy (70 Gy EPBT + weekly cisplatin) axial **(C)** and coronal **(D)** T1-FS contrast-enchanced MRI showing near complete resolution of gross disease.

A recent literature review summarized studies published between 2009 and 2025 and found consistently favorable outcomes with both modalities (**Table 1A**) (34). Since its publication, three additional PBT studies and one CIRT study have further strengthened the evidence base (**Table 1B**) (41–44). Across the proton series, reported 5-year OS ranged from 75% to 86.7%, with local or locoregional control of 84%–98%, while toxicity was generally favorable and predominantly low grade (41–43). A recent CIRT study by Schmich et al. reported 3-year OS and locoregional control of 81.2% and 75.4%, respectively, among patients treated with carbon ions alone or combined photon/CIRT therapy (44). Collectively, these data continue to support the efficacy and acceptable toxicity profiles of both PBT and CIRT in the management of ACC, without demonstrating a clear clinical advantage for either modality.

**Table 1a:** Median patient outcomes from the ACC literature review [Rohit et al].

Series	At last follow-up		Timeframe		
	Local control	Overall survival	Local control	Overall survival	DM
PBT Exclusively ACC	89% (70-100%)	87.5% (54-96.7%)	5-year 88.5% (88-89%)	5-year 76% (59-86%)	25.4%
PBT Mixed	89% (77-95.1%)	67.7% (53-96.1%)	2-year 89% (86-92%)	3-year 77.7% (59.3-96.1%)	20%
CIRT Exclusively ACC	68.6% (67.6-100%)	74.8% (72-100%)	5-year 68.3% (67.6-92%)	74.4% (72-79%)	30%
CIRT Mixed	ACC only: 76.2% (64.6-90%)	86.6% (68-96.8%)	Total: 5-year 69.6% (51-82.9%)	61.2% (47-81.5%)	28.3%

**Table 1b:** Patient outcomes from new literature published after ACC literature review (**Table 1A**) on particle-based radiation therapy for ACC Treatment.

Literature Series	Timeframe	
	Local control	Overall survival
Yacoub et al PBT	5-year LRC 89% Adjuvant: 93% Definitive: 74%	5-year 75% Adjuvant: 86% Definitive: 45%
Vischioni et al PBT	5-year 84%	5-year 86.7%
McGee et al PBT	3-year LRC 98%	3-year 89.2%
Schmich et al CIRT	3-year LRC 75.4%	3-year 81.2%

The literature consistently demonstrates favorable local control (LC) with particle therapy for adenoid cystic carcinoma (ACC) as demonstrated from a recent literature review (**Table 1a**) as well as more recent series published in the interim. These data reinforce the durable efficacy of modern particle-based radiotherapy for ACC. Despite excellent LC, distant metastasis remains a persistent challenge across treatment modalities, occurring in approximately 20–30% of patients.

### Particle therapy access

Patient access to PT remains one of the greatest barriers to widespread implementation. In recent decades, there has been an increased number of particle-based RT treatment centers with PBT treatment centers outnumbering CIRT centers. Currently, there are over 100 PBT treatment centers across the globe with approximately 50 in the US, 30 in Europe, and 30 in Asia (45). Despite the considerable rise compared to previous years, there remains a significant portion of the global population without access to PBT in entire continents such as Africa or Australia without any current operational centers and only one in development in South America. Consequently, many patients must travel long distances to receive treatment, creating substantial financial and logistical barriers that may ultimately preclude access. Patient access to CIRT is more constrained with only 17 centers across the globe with the majority in Japan and China and a few in Europe, specifically, Germany, Italy, and Austria. The first U.S. based CIRT center is set to begin treatment in 2028 (46). Consequently, for many patients world-wide, CIRT is simply not an available treatment option regardless of potential clinical benefits. At the present moment, particle-based RT centers are primarily localized to academic centers in mostly urban communities, limiting access to a significant portion of the general population. In contrast, there are over 8000 XRT treatment centers across the globe with centers available on each continent (47). While this extensive infrastructure makes XRT the most accessible radiation modality, the growing clinical evidence supporting the use of PT highlights an important disparity between technological capability and patient access. As a result, geographic location, institutional referral patterns, and socioeconomic factors frequently influence treatment selection as much as clinical considerations.

### Treatment infrastructure and cost

The limited availability of PT is closely linked to the substantial financial and technical requirements necessary to establish and maintain these facilities. Compared to traditional XRT centers, PBT and CIRT require costly particle accelerators, specialized treatment planning and quality assurance systems, shielding and multidisciplinary expertise (48–51). Capital investment varies considerably according to facility design, number of treatment rooms, and accelerator technology. Contemporary U.S. estimates illustrate the magnitude of this investment, with multi-room proton facilities costing more than approximately US$100–150 million and a proposed US carbon-ion facility estimated in 2020 to require approximately US$300 million in capital investment (52, 53). These estimates vary substantially according to facility configuration and included construction costs. European economic data similarly demonstrate the substantial infrastructure requirements of PT. Peeters et al. estimated 2010 capital costs of €138.6 million for a combined carbon-ion/proton facility, €94.9 million for a proton facility, and €23.4 million for a photon facility, with annual operating costs of €36.7 million, €24.9 million, and €9.6 million, respectively. Treatment costs per fraction were €1,128 for CIRT, €743 for PBT, and €233 for XRT (49). Although published data on facility cost are older, these data provide an internally consistent comparison among modalities but may not reflect contemporary costs associated with newer technologies, inflation, geographic variation, and evolving operational models.CIRT presents additional engineering challenges because carbon-ion delivery generally requires larger and more complex accelerator and beam-delivery systems, shielding, and facility infrastructure (50, 51). These requirements have contributed to the more limited worldwide expansion of CIRT compared with PBT (50, 51). Alternative treatment configurations, such as upright patient positioning and fixed-beam systems, are currently being investigated to potentially reduce treatment-room footprint and system complexity (54).

The higher upfront costs of PT should also be considered in relation to potential long-term clinical benefits and healthcare expenditures. For patients with ACC, who may experience prolonged survival, reductions in late toxicity could potentially translate into downstream healthcare savings. Jensen et al. found that IMRT with a carbon-ion boost increased initial and overall costs compared with IMRT alone, with cost-effectiveness dependent on the assumptions and willingness-to-pay threshold used (55). More broadly, economic evaluations are highly dependent on the perspective adopted, while indirect societal costs and productivity losses remain incompletely represented in available analyses. As PT technology evolves, contemporary studies incorporating capital and operating costs, long-term toxicity, patient outcomes, and societal costs will be important for defining the economic value of these modalities.

### Insurance

Insurance coverage, prior authorization (PA) and insurance denial remain major barriers to patient access to PT, particularly in the United States. Although both PBT and CIRT require substantial institutional investment, reimbursement challenges are more relevant for PBT because of its broader clinical availability. In a retrospective study of 396 patients evaluated for PBT, 64% of those requiring PA were initially denied coverage, and 32% remained denied after appeal despite many meeting the American Society for Radiation Oncology (ASTRO) model policy criteria for appropriate PBT use (56). These denials resulted in treatment delays ranging from 3 weeks to 4 months, potentially compromising oncologic outcomes. Insurers frequently cited the limited availability of randomized phase III evidence, yet insurance denials have also impeded enrollment in such trials, perpetuating a cycle that limits evidence and patient access (56).

Although most published data on insurance barriers originate from the United States, reimbursement challenges vary internationally. In nationalized healthcare systems, access is typically determined by centralized assessments of clinical benefit, cost-effectiveness, and resource allocation rather than individual insurance policies. Regardless of the healthcare model, access to PT remains constrained by infrastructure availability and healthcare economics. As evidence supporting PT continues to grow, reimbursement policies should increasingly consider long-term value rather than upfront treatment costs alone. For patients with ACC, who often experience prolonged survival, reductions in late toxicity, treatment-related morbidity, and secondary malignancies may offset higher initial costs, underscoring the importance of aligning reimbursement policies with contemporary clinical evidence.

## Discussion

From a conceptual point of view, CIRT possesses several compelling theoretical advantages over PBT in the treatment of ACC. Its higher LET and RBE can produce more complex DNA damage, reduce dependence on tissue oxygenation and have the potential for superior efficacy over PBT for relatively radioresistant tumors such as ACC (14, 28–30, 33). However, published clinical outcomes do not support this anticipated advantage (34). Contemporary PBT studies have reported outcomes that are comparable to those observed in CIRT cohorts. These observations should not necessarily be interpreted as evidence of equivalence; they challenge the assumption that higher LET and RBE alone determine the clinical success of ACC treatment. For example, more advanced and unresectable ACC cases are usually treated with carbon ions in respect to PBT series, as reported in Sulaiman et al. and Mizoe et al, making the comparisons of different published cohorts biased (40, 57).

Several factors may explain this apparent disconnect between radiobiologic advantages of CIRT and the lack of reported benefit in clinical outcomes in comparison to PBT.

First, the available evidence is derived almost exclusively from retrospective institutional experiences with substantial differences in institutional clinical experience, patient selection, disease stage, treatment indications, target delineation, dose and fractionation, follow-up duration, and reporting of outcomes (34). These sources of heterogeneity make meaningful cross-study comparisons inherently difficult. Furthermore, due to the limited number of centers worldwide offering both proton and carbon ion therapy, prospective comparative trials are unlikely to be performed for this rare disease.

Second, the excellent outcomes achieved with PBT suggest that factors beyond LET and RBE might contribute substantially to LC. The superior dose conformality afforded by PBT allows reliable delivery of curative-intent radiation doses while minimizing exposure of adjacent OARs (6, 25, 27, 58–68). This advantage is particularly important in ACC, where treatment volumes frequently encompass extensive CN pathways extending to the skull base. Improved target coverage and normal tissue sparing may therefore compensate, at least in part, for the fewer RBE traditionally attributed to PBT.

In addition, the RBE of PBT may be more complex than represented by its conventional clinical RBE of 1.1. Experimental and clinical data have demonstrated increasing LET and RBE near the distal edge of the Bragg peak, with reported values approaching 2 in selected regions (32). Although the clinical implications of this variability remain incompletely understood, these observations suggest that the biologic differences between PBT and CIRT may be less pronounced than traditionally appreciated. Conversely, uncertainties surrounding carbon ion RBE modeling, including differences between the Local Effect Model and the Microdosimetric Kinetic Model, continue to complicate interpretation and comparison of published CIRT outcomes (69).

Finally, differential biological mechanisms of antitumor activity of CIRT versus PBT are far from being understood, and the particle choice in centers with dual beams is mainly based on the stage, margin status after surgery and the histologic subtype. For the future, to maximize treatment outcome in terms of LC improvement with low toxicity burden, biological studies should focus in investigating the biological characteristics of ACC and understanding the antitumor characteristics of different particle type in order to customize particle treatment.

Collectively, these observations and the available literature suggest that the optimal treatment for ACC depends considerably more on the quality of the plan from the dosimetric side than on the characteristic of the particle type. Treatment planning quality, target delineation, dose conformality, institutional expertise, patient selection, and multidisciplinary management likely contribute as much to successful outcomes as differences in LET and RBE. Additionally, since patient access remains the largest barrier to PT treatment selection for diseases such as ACC, the access variable will continue to drive PT selection for patients until equitable access can be achieved.

Future investigation should therefore focus on defining which patients derive the greatest benefit from each radiation modality in order to improve ACC outcome. Comparative dosimetric studies evaluating skull base target coverage and CN irradiation, refinement of biologic dose modeling, multicenter collaborative outcome analyses, and contemporary cost-effectiveness evaluations will all contribute to improving patient selection for PBT vs CIRT. Equally important are continued efforts to expand geographic availability and reduce financial barriers to ensure equitable access to PT worldwide.

## Conclusion

Although CIRT offers compelling theoretical radiobiologic advantages through higher LET and RBE, current clinical evidence has not demonstrated superior outcomes compared with modern PBT. Conversely, the available data are insufficient to establish equivalence between these modalities, given the absence of prospective comparative trials and substantial heterogeneity among published series. Accordingly, current evidence does not support designation of either PBT or CIRT as the preferred PT for ACC. When PT therapy is clinically appropriate, modality selection should be individualized according to target geometry, achievable dosimetry, institutional expertise, and treatment availability. Given the limited global availability of both modalities, particularly of CIRT, patient access remains an important determinant of treatment selection. Future collaborative research should focus on comparative clinical outcomes, biologic modeling, dosimetry, and strategies to expand equitable access to PT.

## Data Availability

The original contributions presented in the study are included in the article/supplementary material. Further inquiries can be directed to the corresponding authors.
